# Update on current pancreatic treatments: from molecular pathways to treatment

**DOI:** 10.7150/jca.36300

**Published:** 2019-08-28

**Authors:** Konstantinos Sapalidis, Christoforos Kosmidis, Varvara Funtanidou, Athanasios Katsaounis, Amastasios Barmpas, Georgios Koimtzis, Stylianos Mantalobas, Vyron Alexandrou, Zoi Aidoni, Charilaos Koulouris, Efstathios Pavlidis, Dimitrios Giannakidis, Valeriu Surlin, Stelian Pantea, Victor Strambu, Rogoveanu Otilia Constantina, Aikaterini Amaniti, Paul Zarogoulidis, Stelian Mogoantă, Isaak Kesisoglou, Chrysanthi Sardeli

**Affiliations:** 1 3rd Department of Surgery, “AHEPA” University Hospital, Aristotle University of Thessaloniki, Medical School, Thessaloniki, Greece; 2 Anesthesiology Department, “AHEPA” University Hospital, Aristotle University of Thessaloniki, Medical School, Thessaloniki, Greece; 3 Department of Pharmacology and Department of Surgery, Faculty of Dentistry, University of Medicine and Pharmacy of Craiova, Craiova, Romania; 4 Clinical Pharmacology, School of Medicine, Faculty of Health Sciences, Aristotle University of Thessaloniki, Thessaloniki, Greece; 5 Surgery Department, University of Timisoara, Romania; 6 General Surgery Department, "Dr Carol Davila", University of Medicine and Pharmacy, Bucuresti, Romania; 7 University of Medicine and Pharmacy, Craiova, Romania

**Keywords:** pancreatic cancer, gene therapy, stem cells, cancer

## Abstract

Pancreatic cancer is still diagnosed at a late stage although we have novel diagnostic tools. Pancreatic cancer chemotherapy treatment resistance is observed and therefore novel treatments are in need. Anti-cancer stem cell therapy, combination of chemotherapy and/or radiotherapy with immunotherapy, proteins/enzymes and gene therapy are currently under evaluation. Targeted treatment with tyrosine kinase inhibitors is also administered and novel inhibitors are also under evaluation. In the current review we present recent data from our search within the year 2018.

## Introduction

Pancreatic adenocarcinoma (PAC) is a common malignancy with very poor prognosis with an overall 5-year survival rate of <5% when all stages are combined. It is also known to have resistance to most chemotherapy regimens 1. Based on current knowledge pre-diagnostic gastrointestinal investigations do not appear to contribute to the poor prognosis of PAC. Currently NICE Guidelines recommend the early use of ultrasound or CT in patients with gastrointestinal symptoms and weight loss. To date we do not have efficient and cost effective screening tests to diagnose this condition at a curable stage and improve survival 2. Moreover; there is a relationship between molecular heterogeneity and clinical features of pancreatic cancer which remains to date unclear. Different subgroups of pancreatic cancer have been identified, which means that different patients in each subgroup might benefit from individual targeted therapy 3. Although different treatments can be used such as; chemotherapy, radiotherapy, immunotherapy and molecular targeted therapy over the past few decades; to date surgical resection is still the most powerful therapy to cure PAC. However; due to lack of early signs or symptoms, most patients are diagnosed at a late stage not amenable to surgery 4. Borderline resectable pancreatic cancer patients treated with neoadjuvant therapy have similar morbidity and survival to their initially resectable pancreatic cancer counterparts 5. It has been observed that modern neoadjuvant therapy is associated with improved survival. However, rapid development of resistance to gemcitabine is always observed 6. Extracellular matrix components have an impact on cell migration and invasive behavior of the cancer cells. Collagen has the most evident effect, and provides new insights into the understanding of the intricate interplay between extracellular matrix molecules and cancer cells. It is a tissue target for novel therapeutic targets for PAC treatment 7. Another type of pancreatic cancer are the pancreatic neuroendocrine neoplasms (PanNENs) which are rare endocrine tumors. It has been observed that they have a different prognosis upon their proliferative state. They are characterized by histopathological grading arising from the endocrine islets with an incidence of 0.2-0.3/100.000 8, 9. The histological classification of these tumors is associated with the Ki-67 index and high mitotic count 10.

Based on the recent WHO classification there are three grades; Grade 1 and Grade 2 tumors which are less proliferative and Grade 3 tumors whose prognosis is very poor 10. Current evidence indicates that neural remodeling and perineural invasion (PNI) of PAC potently increase the risk of cancer relapse, and is considered critical cause of neuropathic pain which affects significantly the quality of life and survival of these patients 11. It has been observed that a 90% of patients are subjected to intra-pancreatic nerve infiltration by cancer cells, and 69% patients have extra-pancreatic nerve involvement 12, 13. Pancreatic nerve infiltration is a common pathological characteristic of PAC where the cancer cells invade the surrounding nerves, which damages the nerve ends 14. Unfortunately to date underlying mechanisms of pancreatic nerve infiltration in PAC are poorly understood. The most important reason for this lack of knowledge is the fact that two third of these patients are diagnosed with pancreatic cancer at a late stage. Therefore, there is an urgent need to develop novel diagnostic tools and therapeutic regimens for pancreatic cancer. In the current article we will provide recent data from 2018 focusing on radiotherapy regimens, molecular targeted treatments, immunotherapy and pancreatic stem cell therapy. Wherever it was necessary we added more data in the text mostly including novel therapies or pathways included in the better understanding of this malignancy.

## Molecular Pathways

### Immunotherapy

**Figure 1:**
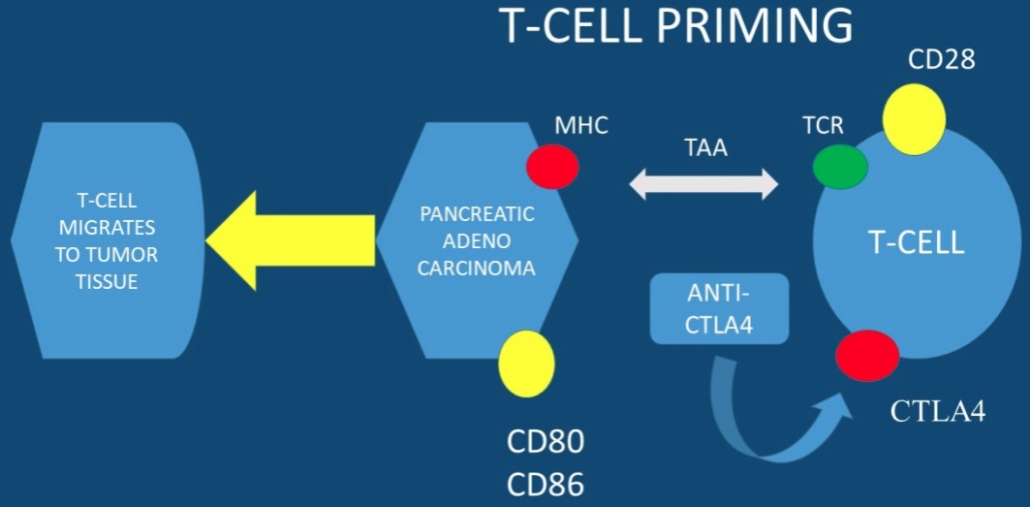
MHC; major histocompatibility complex, TAA; tumor-associated antigens, CTLA4; cytotoxic T-lymphocyte-associated protein 4

**Figure 2:**
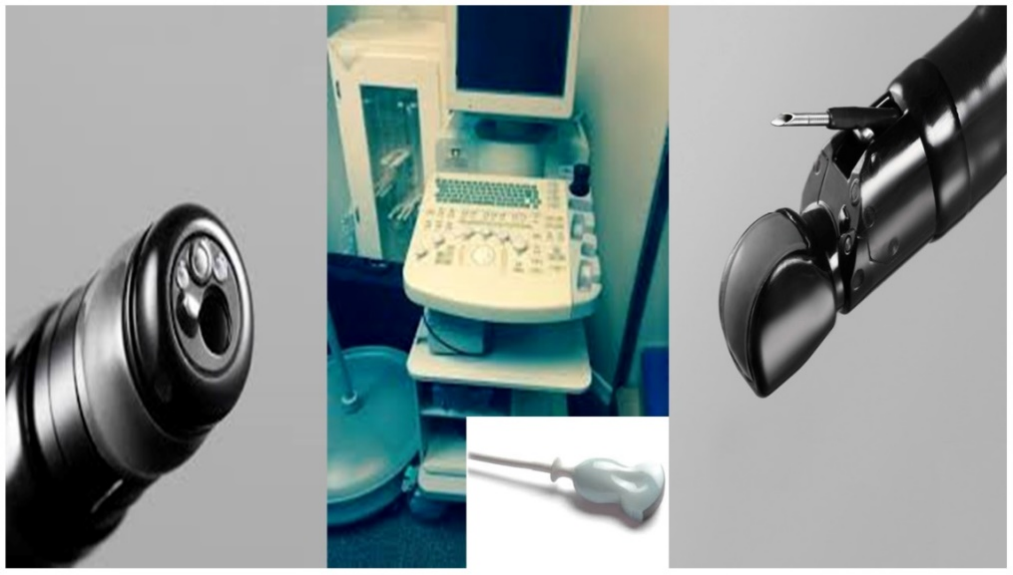
From left to right; Radial esophageal ultrasound, convex probe and convex probe esophageal ultrasound

Currently immune checkpoints blockade therapies, such as; anti-programmed death-ligand 1 (anti-PD-L1) and anti- programmed death-ligand 1 (anti-PD-1), present promising anti-tumor efficacy for several types of solid tumors. However, there are no promising results for this therapy in pancreatic cancer with anti-PD-L1 therapy alone. To date the expression level of PD-L1 is correlated with checkpoint immunotherapy efficacy. It has been observed that esophageal ultrasound fine-needle pancreatic core biopsy (FNB) can be used to determine eligibility for immunotherapy. In a previous study at least 3% of malignant pancreas lesions were sensitive to pembrolizumab and more than 8% were sensitive to the family of immune checkpoint inhibitors. Due to the large EUS-FNB tissue samples precision immuno-oncology can be applied to these patients (PD-L1 evaluation is performed in large tissue samples) 15. The far upstream element-binding protein 1 (FUBP1) is an important transactivator of c-Myc proto-oncogene. FUBP1 has been observed to be overexpressed in pancreatic cancer and is associated with poor prognosis. It has been also observed that FUBP1 promotes tumor cell proliferation and regulates the cancer cell immunity by increasing the PD-L1 expression. FUBP1 is currently investigated as a novel therapeutic modality in order to overcome the immunotherapy resistance in PAC 16. It has observed that PD1-CD28 fusion protein-transduced CD4+ T cells significantly improves anti-tumoral effect of fusion protein-transduced CD8+ T cells. PD1-CD28 fusion protein-transduces CD4+ T cells and has the potential to overcome the PD-1-PD-L1 immunosuppressive axis in pancreatic cancer 17. Combined treatment using radio-immunotherapy with gemcitabine (one cycle) significantly suppresses tumor growth and prolongs survival with tolerable toxicity. Moreover; the two-cycle regimen had the highest anti-tumor effect, but was not tolerable. It was also observed that combination of 90Y-labeled 059-053 and gemcitabine is a promising therapeutic option for pancreatic cancer 18. In the study by Sahin IH et. al. 19 positive results were observed only for the patients with high MSI-H (microsatellite instability) and immunotherapy administration in PAC patients. In another study dendritic cell vaccination was used in order to enhance the FOLFIRINOX regimen and indeed combined treatment significantly increased the lifespan of KIC mice with PAC 20. Avelumab a monoclonal antibody is now being investigated in a phase III clinical trial in combination with PEGPH20 in chemotherapy resistant pancreatic cancer. PEGPH20 (pegvorhyaluronidase alfa) is the PEGylated version of recombinant human hyaluronidase enzyme, rHuPH20. rHuPH20 temporarily degrades hyaluronan (HA), which is a naturally occurring glycosaminoglycan or chain of natural sugars that is common throughout the body and can accumulate in the tumor microenvironment of certain solid tumor types. PEGylating rHuPH20 increases the plasma half-life of the enzyme, which increases exposure following systemic delivery and enables PEGPH20 to target tumor-associated HA in a unique investigational approach to cancer treatment 21, 22. PEGPH20 has a half-life of approximately 2 days, thereby enabling systemic activity and sustained duration of action to degrade HA. In many different tumor types tested in murine xenograft models, response to PEGPH20 has been shown to be more robust for tumors characterized by higher HA expression. The clinical trial NCT03481920 will close in August 2019 (Figure 1,2).

### Pancreatic Stem Cells

**Figure 3:**
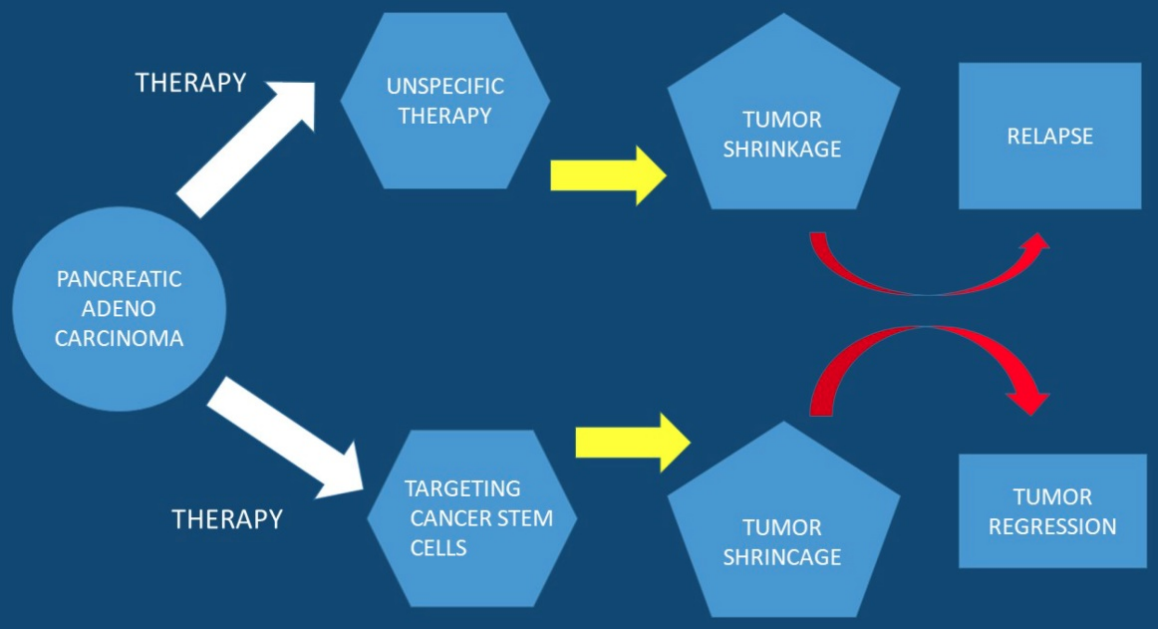
Pancreatic stem cells are used as a targeted therapy

In current studies it has been observed that a subpopulation of cells exist within tumors, cancer stem cell (CSC), which are capable of self-renewal. Cancer stem cells constitute a small cellular subpopulation within the tumor, however; their resistance to chemotherapy and radiation make them an important therapeutic target for treatment. Cancer stem cells possess a unique metabolic plasticity allowing them to rapidly respond and adapt to microenvironmental changes. Cancer stem cells and their metabolic-epigenetic interplay may constitute a new avenue for therapy specifically targeting cancer stem cells in pancreatic cancer 23. Furthermore; metabolic processes of cancer stem cells appear attractive, but due to their function and properties development of resistance is likely to occur. Currently a strategy has been developed to specifically target pancreatic cancer stem cells and it is a phase I/II trial. This trial relies on a different approach by turning the body's immune system against this cellular subpopulation 24. It has been observed that the bioactive sphingolipid induced migration of pancreatic cancer stem cells and signaling was specific to ceramide-1-phosphate. Moreover; pancreatic cancer cells were identified as a rich source of ceramide-1-phosphate. Pancreatic cancer stem cells secrete ceramide-1-phosphate-containing extracellular vesicles as a means of recruiting pancreatic cancer stem cells to sustain tumor growth therefore making ceramide-1-phosphate release a mechanism that could facilitate tumor progression 25. Furthermore; pancreatic cancer stem cell proliferation has been observed to be strongly inhibited by diethyldithiocarbamate-copper complex loaded into hyaluronic acid decorated liposomes. This type of encapsulation of Cu(DDC)2 complex in HA decorated liposomes has been found to strongly increase the proliferative activity of cancer stem cells 26. In another study ``Rauwolfia vomitoria`` extract preferentially inhibited pancreatic cancer stem cells 27. Moreover; ATP Binding Cassette Subfamily G Member 2 which is a non-substrate anticancer agent FL118 has been observed to target drug-resistant cancer stem like cells and overcome treatment resistance of human pancreatic cancer 28. Another agent the BBI-608 has been administered in advanced cancers with positive results and the clinical trial is still ongoing (NCT01775423) 29 (Figure 3).

### Tyrosine Kinase Inhibitors

**Figure 4:**
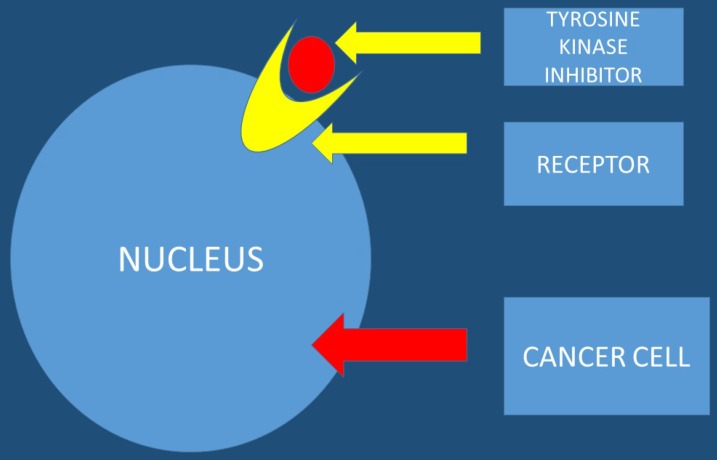
The tyrosine kinase inhibitor blocks the tumor growth

Tyrosine kinase inhibitors are known to be promising anticancer agents. However; resistance is observed in several patients treated with tyrosine kinase inhibitors. The acquired resistance is known to be a complex phenomenon that involves different signaling pathways. Therefore there is a need for drug combination therapies that modulate different signaling and growth systems to counter the emergence of resistance. It is necessary to development multi-targeted tyrosine kinase inhibitors and dual small molecule inhibitors that could improve treatment outcomes and lead to more personalized therapeutic approaches for pancreatic patients 30. Nitroxoline and nelfinavir as single agents or in combination with erlotinib in pancreatic cancer cells can be used 31. Moreover; the EHMT2-p27 axis is a potential marker to modulate cell response to dual PI3K/mTOR inhibition, which might provide a strategy in personalized therapeutics for pancreatic cancer patients 32. This study presented data where the combination of proto-oncogene (RAF) and CDK4/6 inhibitors could be a new treatment strategy for K-ras G12R mutant pancreatic cancer 33. It has been observed that Galunisertib-gemcitabine combination improves overall survival vs. gemcitabine in patients with unresectable pancreatic cancer. To date the use of galunisertib in pancreatic cancer is ongoing in combination with durvalumab (an anti-PD-L1 monoclonal antibody) 34 (Figure 4).

### Enzymes and Proteins

It has been observed that mutant p53 prevents the nuclear translocation of the glycolytic enzyme glyceraldehyde-3-phosphate dehydrogenase. It stabilizes the cytoplasmic localization, and supports glycolysis of cancer cells and inhibits cell death mechanisms by mediating the nuclear enzyme glyceraldehyde-3-phosphate dehydrogenase. The prevention of nuclear localization of enzyme glyceraldehyde-3-phosphate dehydrogenase has been observed to be mediated by both stimulation of AKT and repression of AMPK signaling. Further it is associated with the formation of the SIRT1:GAPDH complex. It has been suggested that mutp53-dependent enhances glycolysis which allows cancer stem cells to acquire sensitivity to anti-glycolytic drugs. Glycolytic enzyme glyceraldehyde-3-phosphate dehydrogenase could be a novel personalized therapeutic approach in human cancers carrying mutant *TP53* gene 35. Hmga2 is known to be a prognostic marker which identifies a metastatic cancer cell state in primary PAC. However; Hmga2 does not have any functional impact on PAC progression and therapy efficiency 36. Integrin b1 is involved in the acquisition of gemcitabine resistance in PAC. Drug-resistance cells from AsPC-1 parent cell line were selected. Results have shown that integrin b1 expression is upregulated in DR-AsPC-1 cells, and integrinb1 knockdown significantly decreases the activity of Cdc42, a target molecule of integrin b1, and p110b expression. It has been observed that knockdown of anyone of integrin b1, Cdc42 and p110b inhibits the activity of PI3K signaling, and sensitizes DR-AsPC-1 cells to gemcitabine. Glutathione-S-transferase pull-down assay has shown that GTP-Cdc42 interacts with p110b. It was observed that integrin b1 promoted gemcitabine resistance in PAC through Cdc42 activation of PI3K p110b signaling. This observation was confirmed by in vivo experiments 37. In another study the role of miR-429 in modulating PNI in PAC was investigated. It was observed that miR-429 was downregulated in PAC cancer tissues and also decreased in tissues with perineural invasion. In and experiment it was reduced in nine out of ten examined pancreatic cancer cell lines. MiR-429 significantly suppresses cell viability and invasion of the pancreatic cancer cells. An online bioinformatic software predicted that neurotrophin-3 (NT-3)has a potential to target the gene of miR-429. It was showed that neurotrophin-3 mRNA elevated in pancreatic cancer tissues in patients with perineural invasion. It has been observed that MiR-429 upregulation substantially suppresses the neurotrophin-3 mRNA andsecretion in pancreatic cancer cells. An interaction between miR429 and NT-3 has been confirmed with dual luciferase reporter assays. MiR-429 potentially suppresses neurotrophin-3 and alleviated perineural invasion of PAC 38.

### Pancreatic Cancer Fibroblasts

Cancer-associated fibroblasts have been observed to be key effector cells in pancreatic ductal adenocarcinoma. They are known to induce tumor growth and progression. Pancreatic stellate cells are the precursors of cancer associated fibroblasts in pancreatic adenocarcinoma that secrete abundant extracellular matrix, cytokines and growth factors. Ηuman relaxin-2 (RLX) which is an endogenous hormone and inhibits pancreatic stem cells differentiation into cancer associated fibroblasts-like myofibroblasts has been evaluated as a therapeutic target. It has been observed that relaxin-2 significantly inhibits tumor growth factor-β and induces pancreatic stem cell differentiation by inhibiting pSmad2 signaling pathway. In specific vitroin primary human pancreatic stem cell, treatment with relaxin-2 dose-dependently inhibited the migration, protein expression of alpha smooth muscle actin, collagen I and contraction. Relaxin-2 has several drawbacks like; poor pharmacokinetics and low systemic vasodilation, which limits its clinical application. A nanoparticle system which chemically conjugates relaxin-2 to superparamagnetic iron oxide nanoparticle (SPION) has been produced to improve its pharmacokinetics. This drug product relaxin-2-spion was observed to be more efficacious compared to free relaxib-2 in vitro. Relaxin-2-spion inhibited tumor growth by itself and also increased the antitumor effect of gemcitabine when injection subcutaneously in a pancreatic tumor stem cell model. In contrast, free relaxin-2 presents no significant antitumor effects. Relaxin-2-spion is an effective antitumor stroma therapy and can be used effectively against pancreatic tumor 39. Cancer associated fibroblasts are known to be highly chemo resistant. Direct cell-cell contact and high levels of interleukin have been correlated with a high chemo resistance 40.

### Proteasome Inhibitors

It is known that inhibition of proteasome activity blocks the degradation of dysfunctional proteins, the result is cancer cell death due to cellular stress. Proteasome inhibitors are a class of anticancer agents, recently; carfilzomib, bortezomib and ixazomib have been FDA-approved to treat multiple myeloma. However, again resistance has been observed to these inhibitors through point mutations in the proteasome ecatalytic subunit. A new proteasome inhibitor was identified aquinolin-chlorobenzothioate, qcbt7, and presents cytotoxicity in a panel of cancer cell lines. QCBT7 is a more stable derivative of quinoline-8-thiol that targets the regulatory subunit. Aquinolin-chlorobenzothioate -7 increases the expression of a set of genes such as; *PFKFB4, CHOP, HMOX1* and *SLC7A11* at both nascent RNA and protein levels. Moreover; the known proteasome inhibitors mg132 and ixazomib do the same. Aquinolin-chlorobenzothioate -7 induces proteasome inhibition, endoplasmic reticulum stress, hypoxic response, glycolysis and cell death. Importantly, 6-Phosphofructo-2-Kinase/Fructose-2,6-Biphosphatase 4 has been identified as a potential biomarker of proteasome inhibitors that can be used to monitor treatment response in pancreatic cancer 41. Mutations or copy number abnormalities of genes involved in homologous recombination (HR) have been previously investigated in PAC tissue samples 29. Pharmacological inhibitors of the enzyme poly ADP ribose polymerase (PARP) are developed for multiple indications; the most important is the treatment of cancer 42. Today PARP inhibitors appear to improve progression-free survival in women with recurrent platinum-sensitive ovarian cancer 43. PARPis strategies target HR repair. Moreover; there are multiple approaches are underway to develop biomarkers for identification of patients who will respond to PARP 44-48. Patients that are cisplatin ineligible may benefit from neoadjuvant use of PARPi combined with carboplatin chemotherapy 49. Adjuvant use of PARPi in patients with high risk or micro-metastatic PAC could potentially render them disease free or be combined with androgen deprivation in node-positive patients 42.

### K-ras

It has been observed that PAC exhibits an oncogenic K-ras mutation rate of ∼90%. Unfortunately to date there is no clinical efficient targeted therapy focused on K-ras mutation PAC. Moreover; gemcitabine resistance is rapidly acquired to these patients. The antibody rt11-i has been created, which directly targets the intracellularly activated GTP-bound form of oncogenic RAS mutants. This antibody significantly sensitizes pancreatic cancer cells to gemcitabine. Furthermore; the co-administration synergistically inhibits angiogenesis, invasion, migration and presents synergistic anticancer activity by inhibiting the RAF/MEK/ERK or PI3K/AKT pathways. Moreover; it was observed that co-treatment inhibits endothelial barrier disruption in tumor vessels, a critical step in vascular leakiness of metastasis, and improves vessel structural stability. The antibody rt11-i synergistically increases the antitumor activity of gemcitabine by inhibiting RAS downstream signaling. In the near future this drug combination could be applied in K-ras positive pancreatic cancer patients 50. G-quadruplex structure is an important drug target in cancer therapy. Porphyrins: Porphyrin-1(Cobalt containing) and Porphyrin-2 (Palladium containing) are observed to have high affinity towards K-ras-promoter/G-quadruplex. These porphyrins exhibited significant cytotoxicity in human pancreatic ductal carcinoma cell line PANC-1 and MiaPaCa2 and blocked metastasis. The mechanism of action is through inhibition of the epithelial to mesenchymal transition. In vivo studies confirmed both porphyrin compounds to be effective against esophageal adenocarcinoma tumors along with significantly low toxicity against normal Swiss albino mice. It has been observed that these porfirins can reduce the expression of K-ras gene in porphyrin-treated PANC-1, tumor-derived esophageal adenocarcinoma and MiaPaCa2 at both protein and RNA levels. Therefore; a porphyrin-based therapy with G quadruplex DNA ligands at the promoter region of K-ras, can be used as an anticancer treatment strategy 51. Moreover; Protein kinase C acts through promoting yes-associated protein 1 function to promote the survival of pancreatic cancer cells expressing *mu-Kras*. This pathway when targeted with YAP1 offers a feasible treatment strategy for developing new therapeutics for treating pancreatic cancer 52.

### MiRNAs

It is known that MiRNAs are small, noncoding RNAs post-transcriptionally regulating gene expression. Several miRNAs with altered expression upon proliferation can be used as prognostic biomarkers in pancreatic neuroendocrine neoplasms. The expression of hsa-miR-106b, hsa-miR-21 and hsa-miR-10a have been observed to have a prognostic relevance regarding progression-free and overall survival in patients with pancreatic neuroendocrine neoplasms 53. Moreover, melatonin and its metabolite N1-acetyl-N2-formyl-5- methoxykynuramine (AFMK) have been observed to enhance chemosensitivity to gemcitabine in pancreatic carcinoma cells (PANC-1). Melatonin and the co-administration of metabolite N1-acetyl-N2-formyl-5-methoxykynuramine can improve the anti-tumor effect of gemcitabine in PANC-1 cells by enhancing the apoptotic pathway 54. Melittin also induced long non-coding RNA proliferation and migration of pancreatic ductal adenocarcinoma 55. It was observed that miR-7/MAP3K9 is critically involved in pancreatic cancer progression and that miR-7 could may be a potential target for pancreatic cancer 56. Moreover; MiR-139-5p transcription is inhibited by EZH2 through up-regulating H3K27me3, thereby downregulation of EZH2 and up-regulation of miR-139-5p impede extracellular matrix and lymph node metastasis in pancreatic cancer. EZH2/miR-139-5p could be a therapeutic strategy for the treatment of pancreatic cancer 57. The amino acid transporter-targeting gemcitabine prodrug, Gem-Thr, was found to be effective on pancreatic cancer cells with increased pharmacokinetic characteristics than gemcitabine alone 58. It has been suggested that S-1 protein could be used as first-line chemotherapeutic option for unresectable pancreatic cancer patients aged ≥75 years 59. SERPINB7 has been identified as the first predictive RNA biomarker for pancreatic cancer. Moreover; patients who expressed SERPINB7 are candidates to receive another treatment than gemcitabine alone 60. In the study by Shahda S et al. 29 mutations or copy number abnormalities of genes involved in homologous recombination (HR) were investigated in tissue samples. The aim of the study was to describe the HR pathway mutations status and determine their association with treatment response and outcome in patients with PAC. However; no positive association was observed between high scores of homologous recombination deficiency or prolonged survival in patients treated with FOLFIRINOX.

### Hypoxia induced factors

There are inhibitors such as the CA9 and APE1/Ref-1that target the hypoxia induced factors and affect pancreatic cancer cell survival 61. A histone deacetylase (HDAC) inhibitor lmk-235 was observed to lower overall cell viability by inducing apoptosisin in a time and dose dependent manner. Acetylation of histone-H3 increases with higher lmk-235 concentrations. Furthermore; IHC analysis showed that proliferative activity (phosphohistone H3 and Ki-67) decreases upon the highest concentrations of lmk-235. Chromogranin and somatostatin receptor 2 (SSTR2) expression increase on the other hand in a dose-dependent manner. Therefore lmk-235 is a potential therapeutic approach pancreatic neuroendocrine neoplasms 62, 63. Metarrestin is an effective therapeutic inhibitor and a candidate with a favorable pharmacokinetic profile achieving excellent intra-tumor tissue levels in pancreatic cancer. Pharmacokinetic evaluation of metarrestin in wild-type and Pdx1-Cre;LSL-K-rasG12D/+;Tp53R172H/+ (KPC) mice, showed significant antitumor activity 64.

### Radiation

Currently there is a debate of dosimetric analysis of stereotactic rotational versus staticintensity-modulated radiation therapy for PAC. Nine-field intensity-modulated radiation therapy reduces the number of monitoring units and treatment delivery time when compared to volumetric-modulated arc therapy. Volumetric-modulated arc therapy is efficient for locally advanced PAC if combined with SBR 65. Novel 4D-MRI sequence based on 3D-radial sampling and slab-selective excitation observed that the non-contrast 4D-MRI images showed significantly better contrast when compared to noise ratio for the vessels. The specific sequence is more efficient in identifying both the tumor and boost volume margins for pancreas radiotherapy 66. Moreover; It was observed that stereotactic body radiotherapy and anti-CD40 are so effective at augmenting T cell priming, that memory CD8 T cell responded that both tumor and self-antigens were induced, resulting in vitiligo in long-term survivors 67. In another study the SCAD score was evaluated as a method to identify individuals benefiting from re-stereotactic body radiotherapy 68. PARP inhibitors can be used in combination with chemo- or radiotherapy or as single agents alone 69.

Finally in locally advanced it has been observed that there is a higher effectiveness when chemoradiotherapy is applied simultaneously for locally advanced pancreatic cancer 70. Regarding borderline resectable patients then it has been observed in a Phase II trial that preoperative FOLFIRINOX followed by individualized chemoradiotherapy in borderline resectable pancreatic cancer results in high rates of R0 resection and prolonged median PFS and median OS, supporting ongoing phase 3 trials 71.

## Future perspective

Currently the major issue is to predict the response and to identify additional targets that will improve the efficacy of chemotherapy of pancreatic cancer. In another study it was initiated through a large-scale in vivo and in vitro CRISPR knockout screens in pancreatic ductal adenocarcinoma cells. It was observed that several gene deletion synergistically increased the cytotoxicity of MEK signaling inhibitors. Drugs were created based on in vivo CRISPR screening (DREBIC) method and validated their efficacy using large-scale experimental data from independent experiments. It was observed that comparative analyses demonstrated that DREBIC predicts drug response in cancer cells from a wide range of tissues with high accuracy 72. There are currently oncolytic viruses for pancreatic cancer (current data and clinical trials) 73. It is known that oncolytic viruses have been used as a novel class of anti-cancer therapeutics with one virus already receiving United States Food and Drug Administration (FDA) approval (talimogene laherparepvec). It has been observed that these viruses have direct lytic effects on tumor cells as well as immunomodulatory functions which increase the inflammatory cell infiltrates in the tumor microenvironment. However; despite all of the advances this therapeutic modality still remains inefficient. One of the main reasons is the fibrotic tumor stroma and the unique extracellular matrix which creates an environment that promotes tumor growth. An oncolytic virus is a virus that preferentially infects and kills cancer cells. As the infected cancer cells are destroyed by oncolysis, they release new infectious virus particles or virions to help destroy the remaining tumor. Oncolytic viruses are thought not only to cause direct destruction of the tumor cells, but also to stimulate host anti-tumor immune system responses.

A number of viruses including adenovirus, herpes simplex, reovirus, measles, and vaccinia have been clinically tested as oncolytic agents. Most current oncolytic viruses are engineered for tumor selectivity, although there are naturally occurring examples such as reovirus and the senecavirus, resulting in clinical trials 73.

There are underlying pathologic correlations with typical and atypical presentations of pancreatic neuroendocrine neoplasms 74. Liquid biopsy could be a tool to assess potential biomarkers. However; the method still has setbacks and tissue is still absolutely necessary 75. Currently the method of liquid biopsy is efficient 60-70% for identifying mutations 76. Moreover; further prospective trials are needed to define the optimal time-dose-fractionation (radiotherapy) in order to reduce toxicity and improve the palliative outcome. Furthermore; pain control and quality of life should be considered as an endpoint in future trials with local treatment (advanced stage disease 77. Functionalized MoS2 Nanosheets as Multi-Gene Delivery Vehicles for *In Vivo* Pancreatic Cancer Therapy have been also constructed to carry gene therapy 78. In specific the potential of this polymer to encapsulate and enhance drug (5-fluorouricil and bis-(naphthalimidopropyl)-diaminooctane) cytotoxicity in BxPC-3 cells was evaluated. Furthermore; the novel poly(allylamine)-naphthalimide carrier it was observed to amplify the cytotoxic effect with drug treatment after 24 h. The result was reduction of 50% of cancer cell population 79. Monitoring quality of care for patients with pancreatic cancer was performed with novel quastionaires such as the modified Delphi consensus 80. There are novel circulating nucleic acids that are associated with outcomes of patients with pancreatic cancer 81. There is a novel local therapy in a Phase I Study of EUS-guided photodynamic therapy for locally advanced pancreatic cancer 82. There is currently a debate for evaluation of the prognostic value of the new AJCC 8^th^edition staging system for patients with pancreatic adenocarcinoma, since there is a need to sub classify stage III?^83^. Currently we have radiofrequency ablation as a treatment option for liver cancer with significant results. On the other hand the use of radiofrequency ablation is relatively new in PAC. Radiofrequency ablation is a promising mechanism to induce antigen-presenting cell infiltration and enhance systemic antitumor T-cell immune response and tumor regression as observed with other solid tumors. Combination of radiofrequency ablation with immunotherapy could represent a novel and promising treatment 84. To date several factors have been related to the status of para aortic lymph nodes such as; preoperative CA19-9 level, portal vein/superior mesenteric vein invasion, superior mesenteric artery invasion and diameter>1.0cm were 4 independent risk factors to para aortic lymph nodes metastasis. It has been also observed that positive LN8 and LN14 are 2 strong predictors of para aortic lymph nodes metastasis. Therefore a comprehensive analysis covering these risk factors related to metastasis of para aortic lymph nodes should be given before design of future treatment plan whenever involvement of para aortic lymph nodes is suspected 85. Finally, we have to introduce the older cancer patient to patient activation through counseling, exercise and mobilization 86.

## Conclusion

Immune checkpoint inhibitors are a treatment choice for several cancer types and have shown a certain efficacy in the treatment of advanced pancreatic cancer. Therefore could confer long-term survival benefits when used. Combined therapy when possible is certainly more effective and may serve as an alternative option. Further studies should be performed towards combined therapies 87. Combination systemic therapies or radiotherapy with immune checkpoint inhibitors in pancreatic cancer can overcome resistance to single‑agent checkpoint blockade 88. Radiotherapy of pancreatic cancer in older patients is feasible and can be used. Radiotherapy can represent a treatment option in pancreatic cancer even in older patients. However; further analyses and prospective trials enrolling older patients are needed to better define the risk/benefit ratio in different treatment settings (e.g performance status, metastatic site) 89. Association between chronological depressive changes and physical symptoms in postoperative pancreatic cancer patients is important for treatment administration and should be evaluated before treatment administration 90.
